# A medical student perspective on sexual history-taking: doing it for the first time

**DOI:** 10.1080/10872981.2018.1447206

**Published:** 2018-03-06

**Authors:** Vignesh Gopalan, Surina Taneja

**Affiliations:** ^a^ Medical School, University College London, London, UK

A sexual history offers an opportunity to identify risk factors for contracting sexually transmitted infections and to promote safe sexual practice, thus is an important skill for healthcare professionals regardless of specialty. However, despite medical students and clinicians alike agreeing that taking a sexual history is an important skill, many feel ill-prepared to take one [1,2]. We present our own experiences of sexual history-taking and discuss how its teaching can be improved in medical schools.

Embarrassment, fear of being insensitive and cultural differences can make sexual history-taking a challenge for any medical student or clinician [2]. This applies even more so to those with inadequate training. A government report suggests this failure of clinicians to address sexuality and sexual health in consultations stems from an ‘inadequate, patchy or absent sexual health training in undergraduate curricula’ [3]. A survey by the University of Bristol medical school of 22 UK medical schools shows that the mean time dedicated to teaching sexual history-taking is 1.8 h, whereas students spend 6.8 h in genito-urinary medicine clinics [4]. From our experience, watching clinicians may not be the most effective way to acquire and practice the skills needed to conduct a sexual health consultation.

Asked to take our first sexual histories in a clinic, we found it difficult to phrase sensitive questions and were worried about embarrassing ourselves (or worse, the patient). Hesitance led us to omit certain topics entirely. On reflection, although we feel that taking histories under supervision is an integral part of student training, additional techniques implemented in other aspects of our clinical education may allow us to make more of this opportunity. For example, performing intimate examinations on patient-experts made us more confident prior to seeing patients and the use of group role-play has allowed us to identify examples of good practice and potential pitfalls when having difficult discussions. An initiative combining group work, role-play and clinical attachments was found to encourage discussion on attitudes and assumptions surrounding sexual behaviour [4]. Further review of the literature shows that students report greater comfort in sexual history-taking after mock consultations [5] and that students who train using a multi-modal approach to sexual history-taking perform better in exams [6].

Following on from student feedback, our medical school is introducing simulated teaching sessions into our sexual health curriculum. We hope that establishing this form of teaching has the potential to shape a generation of medical students more competent and comfortable in identifying sexual health issues.
